# Things Spiritual and Economic

**Published:** 1918-11-09

**Authors:** 


### THE NURSES' DAY AND FUTURE OUTLOOK.

### Things Spiritual and Economic.

Without homilising or entering the domain of platitude, it may be useful to mention that nursing has its spiritual side. The "ministering angel" is something more than a person skilled in the healing art, something greater than the mechanical interpreter of doctors' wishes, expressed and understood.

"She nurses best who always has in mind
That touch so tender and that look so kind
Of Him Who came this mortal frame to wear.

. . . . . . .

The hands that thus do serve have wondrous Love,
They never weary, never careless prove;
To such the night watch, silent as the tomb
Save for its groans, has neither dread nor gloom.
No task too menial, naught too hard can prove,
Their meanest act is sanctified by Love.
Nurse on, dear daughter, shrink not, it is He
On the white throne shall say 'It was for Me.'"

Unfortunately, she who "nurses best," according to the standard of the Dean of Exeter, is not present in every hospital ward. With many excellent qualifications there is too often yet one thing needful.

Florence Nightingale, always practical and plain-spoken, expressed the same sentiment otherwise. Her requirements were exacting in the extreme. May I quote a few of the most elementary and general?

"Go your way straight to God's work in simplicity and singleness of heart."

"The very alphabet of a nurse is to be able to interpret every change which comes over a patient's countenance without the exertion of saying what he feels."

"A nurse must be something more than a lift or a broom. A patient is not merely a piece of furniture to be kept clean and ranged against the wall, and saved from injury or breakage—though to judge from what many a nurse does or does not do you would say he was."

"What is it to feel a 'calling' for anything? Is it not to do your work in it to satisfy your own high idea of what is the right, the best, and not because you will be found out if you don't do it? This is the enthusiasm which everyone, from a shoemaker to a sculptor, must have in order to follow his calling properly. . . . A nurse who has not such a calling will never be able to learn the sound of her patient's bell from that of others."

"A good nurse scarcely ever asks a patient a question—neither as to what he feels nor as to what he wants. But she does not take for granted, either to herself or others, that she knows what he feels and wants without the most careful observation and testing of her own observations."

I might go on quoting the words of this wonderful woman whose prescience and wisdom were of the highest, but what I have given is probably sufficient to show that Florence Nightingale expected every nurse to give her soul as well as her body and her intellect to her work. I have called this, for want of a better name, the spiritual side of nursing, and I have entered on this phase of the subject with a distinct purpose. Writers have recently endeavoured to explain the absence or the disappearance of this essential quality, and some have described it as the "hardening" of the nurse as she becomes experienced. But, so far as I am aware, none of these has discovered the true cause, which is to be found absolutely and altogether in the economic conditions of her employment. The nurse is the product of her environment, and she is no more to be blamed for "hardening" than a rose is to be blamed for fading in a corner where there is no sun. Sordid surroundings produce sordid natures. If you want to find beautiful character you do not seek for it in "the crowded warrens of the poor." Here criminals are bred. Spiritual natures are not independent of physical conditions: enthusiasm is the correlative of health, and I am as convinced as I can be of anything that spiritual impulse can be destroyed and wholesome enthusiasm crushed by harsh conditions of life.

The nursing of the nation's sick, according to Florence Nightingale, is God's work. From a lower plane I desire to emphasise that it is the nation's work, and to ensure that it is performed according to the highest standard is a public duty. There is no more arrogant and ignorant attitude, therefore, than the "mind-your-own-business" of some of the ladies of the "London" and other correspondents of this paper who live in the delusion that the public hospital is their property, and who think that if they themselves are satisfied the interference of "outsiders" is an impertinence. I want such as these to lift their heads out of the sand and look around. The nursing profession is not on the up-grade, and the British public have a right to know the truth. The harshness and bondage of the nurse's life are having the dual effect of preventing the best class of recruits from entering, and of crushing the souls of those who do. No bogus Charters of Liberty or cheap appeals to patriotism can prevent the natural laws that govern human life from fulfilling their immutable purpose. The nurse's life is not to be ennobled by process of preaching, but by material benefit. Give her a career, a goal of honour if you please, but also a goal of comfort. I was horrified recently to read an honest answer to an inquiry made by an industrious and successful nurse whose work was done as to how she might provide for the future. The answer was, If possible, to find admission to an almshouse, and it was pointed out that even this door of hope is difficult of entrance. The situation is not merely pathetic: it is tragic. Possibly an almshouse, or something worse. This is one outlook of the British nurse, and when Major Chapple complained that her earnings are being absorbed in hospital revenues, Lord Knutsford reproaches him with being a New Zealander and an "honourable" M.P. who knows nothing about what he is talking, while some of his lordship's satellites retort "Mind your own business!"

What is wanted is a bold campaign of propaganda. Florence Nightingale is dead, but her spirit lives. Her bequest to humanity was the Trained Nurse, the nation's most valuable asset. The cause of the Trained Nurse must be championed and her claims rung in the ears of the British public. The hospital must be lifted out of the slough of poverty, and the women who work therein treated as honoured members of the community. A cheap halo during service and an almshouse, possibly a workhouse afterwards, are not the factors that make for high ideals, enthusiasm, efficiency, and spiritual devotion.

IERNE.

